# *CD40* polymorphisms in Han Chinese patients with Fuch uveitis syndrome

**Published:** 2011-09-23

**Authors:** Feilan Chen, Shengping Hou, Zhengxuan Jiang, Fuzhen Li, Yuanyuan Chen, Aize Kijlstra, Peizeng Yang

**Affiliations:** 1 The First Affiliated Hospital of Chongqing Medical University, Chongqing Key Laboratory of Ophthalmology, and Chongqing Eye Institute, Chongqing, P.R. China; 2 Eye Research Institute Maastricht, Department of Ophthalmology, University Hospital Maastricht, Maastricht, the Netherlands

## Abstract

**Purpose:**

Polymorphisms of the cluster of differentiation 40 (*CD40*) gene have recently been identified to be associated with the risk to several immune diseases. The aim of this study was to determine the potential association of *CD40* polymorphisms with Fuch uveitis syndrome (FUS).

**Methods:**

A total of 131 Han Chinese patients with FUS and 402 healthy controls were genotyped using the polymerase chain reaction-restriction fragment length polymorphism (PCR-RFLP) method. Genotype counts in patients and controls were analyzed by the χ^2^ test.

**Results:**

All genotypic and allelic frequencies of the tested two *CD40* polymorphisms were in Hardy–Weinberg equilibrium. The genotypic and allelic frequencies of rs4810485 and rs1883832 were not different between patients with FUS and controls. No influence of sex could be found following stratification analysis according to gender.

**Conclusions:**

Our results suggest that the two investigated single-nucleotide polymorphisms (SNPs), rs4810485 and rs1883832, in *CD40* are not associated with FUS in the Han Chinese population.

## Introduction

Fuchs uveitis syndrome (FUS) is a chronic unilateral ocular inflammatory disease which mainly affects young adults. It is a relatively common uveitis entity in China and is characterized by medium-sized or stellate keratic precipitates, a variable degree of iris depigmentation or heterochromia without posterior synechiae [1]. Although the etiology of FUS has not yet been fully elucidated [2], both environmental and genetic risk factors have been suggested to be involved in its pathogenesis. Several groups have identified an association with rubella infection and FUS [3] and a decline has been noted in the incidence which is ascribed to vaccination programs against rubella. Earlier studies showed an association of the human leukocyte antigen-B18 (*HLA-B18*) with FUS [4]. Furthermore, recent studies have addressed the association of polymorphisms of the intercellular adhesion molecule 1 (*ICAM-1*) gene [5], the cytotoxic T cell antigen (*CTLA*) *4* gene [6], and the interleukin 23 receptor (*IL23R*) gene [7] with this disease.

The cluster of differentiation 40 (*CD40*) gene is located on chromosome 20q13 and encodes the CD40 protein, which plays an important role in the onset and severity of multiple inflammatory autoimmune diseases and the initiation and maintenance of inflammation triggered by infections through interaction with its ligand (cluster of differentiation 154 [CD154]) [8]. Recently, genome-wide association surveys have identified the association at the *CD40* locus with rheumatoid arthritis [8] and multiple sclerosis [9]. Multiple candidate gene studies have also identified and validated the association of several SNPs in *CD40* with autoimmune or autoinflammatory diseases including Graves’ disease [10], Crohn's disease, multiple sclerosis [11], and rheumatoid arthritis [12]. As CD40-CD40L binding is required in the priming of cluster of differentiation 8^+^ (CD8^+^) T cell following virus infection, the *CD40* polymorphism might influence the cytotoxic T cell responses in the virus infection associated diseases, such as FUS which has been reported to be associated with rubella and CMV infection. Therefore, we investigated the association of rs4810485 and rs1883832 at the *CD40* locus with FUS. Unfortunately, we failed to find any association of its polymorphisms with this disease in a Han Chinese population.

## Methods

### Patients and healthy controls

A total of 131 Han Chinese patients with FUS and 402 age-, sex-, and ethnic-matched healthy controls were consecutively recruited from the Uveitis Study Center of the Sun Yat-sen University (Guangzhou, P.R. China) and the First Affiliated Hospital of Chongqing Medical University (Chongqing, P.R. China). The diagnosis of FUS was made according to the clinical manifestations as described earlier [1] Briefly, the clinical manifestations include low-grade anterior chamber response, characteristic medium-sized or stellate keratic precipitates, and diffuse iris depigmentation or heterochromia without posterior synechiae. The Ethics Committees of the First Affiliated Hospital of Chongqing Medical University approved the study. The tenets of the Declaration of Helsinki were conducted during all procedures of this present study. The donors were enrolled in the study after informed consent.

### Genomic DNA extraction and genotyping

Blood samples were collected in EDTA tubes and kept at −70 °C until use. Genomic DNA was isolated using the QIAamp DNA Blood Mini Kit (Qiagen, Valencia, CA ). The primers of the *CD40* gene polymorphisms listed in Table 1 were used to amplify the target DNA by the polymerase chain reaction (PCR). Each PCR reaction was conducted in 10 μl containing 5 μl Premix Taq (Ex Taq Version; TaKaRa Biotechnology Co. Ltd., Dalian, China), 20 pmol primers, and 0.2 μg of genomic DNA for amplification of the DNA. The conditions were as follows: initial denaturation at 95 °C for 5 min followed by 38 cycles of denaturation at 95 °C for 30 s, annealing at 58 °C for 30 s, extension at 72 °C for 25 s, and a final extension at 72 °C for 5 min. The two tested SNPs were genotyped by PCR-restriction fragment length polymorphism (RFLP) analysis. PCR products of the rs4810485 and rs1883832 polymorphisms were respectively digested with 4 U of MspI (Fermentas International Inc., Burlington, Canada) and NcoI (Fermentas International Inc.; Table 1) in a 10 μl reaction volume overnight. The amplified PCR products were electrophoresed on a 3.5% agarose gel and stained with GoldView™ (SBS Genetech, Beijing, China). The results were validated by direct sequencing by the Majorbio Bio-Pharm Technology Company (Shanghai, China) in 10% of the total samples.

**Table 1:** Primers of CD40 SNPs and restriction enzymes for RFLP analysis

**Rs number**	**Primers**	**Restriction enzyme**
rs4810485	5′-TATTTTTGTAGTTCCTCATTCTG-3′	MspI
** **	5′-GCCCCCCTTTACCTCTTTC-3′	** **
rs1883832	5′-CCCCGATAGGTGGACCGCGATTG-3′	NcoI
** **	5′-CCCGCCCTCTGAACCCCCTACCA-3′	** **

RFLP, restriction fragment length polymorphism.

### Statistical analysis

Hardy–Weinberg equilibrium (HWE) was evaluated in the subjects using the chi-square test. Genotype frequencies were obtained by direct counting. The statistical analysis was performed using the χ^2^ test with SPSS (version 17.0; SPSS Inc., Chicago, IL) to compare the genotype and allele frequencies between patients and controls. We used Bonferroni correction to account for multiple testing. The statistical significance was set at pc<0.05.

## Results

One hundred and thirty-one consecutive Han Chinese patients with FUS were enrolled in the present study (72 males and 59 females). The average age of the patients with FUS in the present study was 38.2±10.1 years (age range 16–68 years). The healthy controls consisted of 402 subjects (241 males and 161 females), in which the average age was 37.2±10.6 years (age range 12–72 years). The age and gender distribution of the patients with FUS and controls are summarized in Table 2.

**Table 2:** Age and gender distribution in Fuchs’ patients and controls.

** **	**Patients with FUS**		**Healthy controls**	
**Clinical features**	**Total (n=131)**	**%**	**Total (n=402)**	**%**
Age (mean ±SD)	38.2±10.1	** **	37.2±10.6	** **
Range	16–68	** **	12–72	** **
**Sex**				
Male	72	55	241	60
Female	59	45	161	40

FUS: Fuchs uveitis syndrome.

Two single-nucleotide polymorphisms (SNPs) of *CD40* (rs4810485 and rs1883832) were successfully genotyped in 131 patients with FUS and 402 healthy controls. The results showed that the distribution of genotypes and alleles in subjects were in HWE. The distribution of genotypic and allelic frequencies of the tested two *CD40* polymorphisms is presented in Table 3. A trend of increased frequency of genotype of rs4810485 TT and rs1883832 TT was observed in the Fuchs’ patient cohort as compared with controls. However there was no statistically significant difference concerning the genotype and allele of both rs4810485 and rs1883832 SNPs between Fuchs’ patients and controls. In addition, we failed to find any association of the tested gene polymorphisms with FUS by stratification analysis according to gender.

**Table 3:** Allelic and genotypic distribution of *CD40* polymorphisms in Fuchs’ patients and controls.

**SNP**	**Genotype allele**	**Fuchs’ patients (n=131)**	**Controls (n=402)**	**χ^2^**	**p value**	**Pc**	**OR (95% CI)**
rs4810485	TT	27 (20.6%)	60 (14.9%)	2.338	0.126	NS	1.48 (0.89–2.45)
** **	GT	59 (45.0%)	197 (49.0%)	0.623	0.43	NS	0.85 (0.57–1.27)
** **	GG	45 (34.4%)	145 (36.1%)	0.127	0.721	NS	0.93 (0.61–1.40)
** **	T	113 (43.1%)	317 (39.4%)	1.125	0.28	NS	1.10 (0.92–1.33)
** **	G	149 (56.9%)	487 (60.6%)	1.125	0.28	NS	0.29 (0.75–1.09)
rs1883832	TT	26 (19.8%)	67 (16.7%)	0.296	0.586	NS	0.87 (0.52–1.44)
** **	CT	61 (46.6%)	193 (48.0%)	0.083	0.774	NS	0.94 (0.64–1.40)
** **	CC	44 (33.6%)	142 (35.3%)	0.131	0.717	NS	0.93 (0.61–1.40)
** **	T	113 (43.1%)	327 (40.7%)	0.493	0.482	NS	1.11 (0.83–1.47)
** **	C	149 (57.5%)	477 (59.3%)	0.493	0.482	NS	0.90 (0.68–1.20)

Pc Bonferroni corrected p value. NS=Not significant. OR=odds ratio. 95% CI=95% confidence interval.

## Discussion

No association was found between the rs4810485 and rs1883832 polymorphisms of *CD40* with FUS in a Han Chinese population.

CD40 is a T cell membrane protein which is critical for CD8^+^ T cell differentiation and the CD8^+^ T cell-mediated response [13-15]. CD40 is expressed on antigen-presenting cells (APCs) such as macrophages and B cells. CD40 stimulation contributes to APC activation and is required for the priming of CD8^+^ cytotoxic T cell (CTL) and CD4-cell dependent CTL responses to some viruses through interaction with CD154. The interaction of CD40 and CD154 have been shown to play a role in various diseases such as autoimmune thyroiditis, type 1 diabetes, inflammatory bowel disease, psoriasis, multiple sclerosis, rheumatoid arthritis, and systemic lupus erythematosus [16]. Earlier studies have shown that CD8^+^ T cells are predominant cells in the aqueous humor in patients with FUS [17], suggesting a possible involvement of CD40 in the pathogenesis of this disease.

As there are many SNPs in a candidate gene and only few SNPs may contribute to the disease, it is very important to determine how many and which SNPs should be selected as optimal candidates within the gene to test an association with disease. In the present study, we chose rs4810485 and rs1883832 as the tested SNPs because these were also used in earlier studies [8,11]. rs4810485 was shown to be associated with rheumatoid arthritis risk in Caucasians [8], but not in patients with a Korean ancestry [18]. Our findings showing that rs4810485 was not associated with FUS could therefore also be due to the ethnic background of our patients.

We also investigated rs1883832, whereby the common variant increases the efficiency of CD40 translation and which has been associated with Graves’ disease [19,20], multiple sclerosis, and Crohn’s disease [12]. Of interest is the observation that the minor allele rs1883832 T is protective in Graves’ disease but enhances risk of disease in Crohn’s disease and multiple sclerosis [12]. We were not able to demonstrate an association between rs1883832 polymorphisms and FUS.

Associations between CD40 polymorphisms have mainly been shown in autoimmune diseases and the absence of an association with FUS may be due to its infectious etiology [16,21]. Recent studies have for instance detected rubella virus in the anterior chamber and have shown an increased intraocular rubella antibody production in these patients [22].

Since association studies may be influenced by numerous factors, the following measures were used to validate the results. The healthy controls and patients were strictly age-, sex-, and ethnically matched. To validate the result of genotyping by PCR-RFLP, 10% of samples were directly sequenced.

It is worthwhile to point out that there are some limitations in the present study. The sample of the patients was relatively small and only Han Chinese cohorts were included. Therefore, our study is relatively under-powered to examine an association with Fuchs’ patients. Larger sample size is needed to detect the association of *CD40* with this disease. Although we failed to find any association between FUS and the investigated two SNPs which have been shown to be associated with several diseases, our result does not rule out the possibility that other SNPs of *CD40* are associated with this disease. Therefore, study is needed to clarify the association of other SNPs of *CD40* with this disease.
